# Baicalin Induces Apoptosis and Suppresses the Cell Cycle Progression of Lung Cancer Cells Through Downregulating Akt/mTOR Signaling Pathway

**DOI:** 10.3389/fmolb.2020.602282

**Published:** 2021-01-28

**Authors:** Xinbing Sui, Xuemeng Han, Peng Chen, Qibiao Wu, Jiao Feng, Ting Duan, Xiaying Chen, Ting Pan, Lili Yan, Ting Jin, Yu Xiang, Quan Gao, Chengyong Wen, Weirui Ma, Wencheng Liu, Ruonan Zhang, Bi Chen, Mingming Zhang, Zuyi Yang, Na Kong, Tian Xie, Xia Ding

**Affiliations:** ^1^School of Traditional Chinese Medicine, Beijing University of Chinese Medicine, Beijing, China; ^2^Department of Medical Oncology, School of Medicine, The Affiliated Hospital of Hangzhou Normal University, College of Pharmacy, Hangzhou Normal University, Hangzhou, China; ^3^Key Laboratory of Elemene Class Anti-Cancer Chinese Medicines, Engineering Laboratory of Development and Application of Traditional Chinese Medicine, Collaborative Innovation Center of Traditional Chinese Medicines of Zhejiang Province, Hangzhou Normal University, Hangzhou, China; ^4^State Key Laboratory of Quality Research in Chinese Medicines, Faculty of Chinese Medicine, Macau University of Science and Technology, Macau, China

**Keywords:** baicalin, cell cycle, apoptosis, lung cancer, Akt

## Abstract

Baicalin, as a natural active ingredient extracted and isolated from the traditional Chinese medicine *Scutellaria baicalensis* Georgi., has been potentially used in various areas for its antioxidative, antitumor, anti-inflammatory, and anti-proliferative activities. Although several studies have reported the antitumor effects of baicalin against various cancer types, its beneficial effects on lung cancer have not yet been elucidated. Therefore, the therapeutic effects and molecular mechanisms of baicalin on lung cancer cell lines H1299 and H1650 were investigated. Here, the results of its antitumor activity were shown. We found that Akt/mTOR pathway inhibition was the essential determinant in baicalin-induced cell cycle arrest. Furthermore, when the Akt Agonist SC79 or Akt plasmid transfection was performed, the antitumor effect of baicalin was significantly abrogated in both H1299 and H1650 cells. In conclusion, we found that baicalin exerted its antitumor activity mainly by inducing Akt-dependent cell cycle arrest and promoting apoptosis, which show great potential for developing a new drug for lung cancer treatment.

## Introduction

Lung cancer is still one of the most fatal cancers in the world (Bray et al., 2018). Surgery, chemotherapy, radiotherapy, and molecular target therapy are major treatment options for lung cancer patients (Fiero et al., 2019). Despite recent advances in new antitumor agents and intensity-modulated radiation therapy, the side effects and toxicity of these strategies have produced a bottleneck in clinical lung cancer treatments (Hellmann et al., 2019; Crunkhorn, 2020). Therefore, there is an urgent need to discover alternative reagents or novel therapeutic approaches for lung cancer patients.

Natural compounds have always been one of the most important sources of antitumor drug discovery. Baicalin, which is isolated from the *Scutellaria baicalensis* Georgi (Huang Qin), has been clinically utilized for centuries as a traditional Chinese herbal medicine in China. Baicalin was shown to display various biological properties, including antioxidative, anti-proliferative, anti-inflammatory, antitumor activities, and protective effects against multiple-tissue or organ damage (Chen et al., 2014; Fu et al., 2020; Liu et al., 2020; Song et al., 2020). Recently, few studies have revealed the antitumor action of baicalin in lung cancer cells (Wei et al., 2017; Diao et al., 2019); however, the therapeutic effects and underlying mechanism of baicalin in lung cancer have not been elucidated yet.

In this research, the antitumor activity of baicalin was investigated on the two different lung cancer cells H1299 and H1650, and the results show that baicalin could induce apoptosis as well as cell cycle arrest of lung cancer cells, indicating that baicalin has multiple antitumor mechanisms. According to our study, baicalin was proved for the first time to inhibit the Akt/mTOR pathway, which mainly contributed to the cell arrest and survival inhibition, for its employment in lung cancer treatment. Furthermore, Akt agonist SC79 or Akt overexpression plasmid transfection abrogated the antitumor effect of baicalin in these two lung cancer cells. In a word, our results suggested that baicalin exerted its antitumor activity by promoting apoptosis and inducing Akt-dependent cell cycle arrest in lung cancer cells.

## Materials and Methods

### Cell Culture and Transfection

The human lung cancer H1650 and H1299 cell lines were purchased from ATCC, which were cultured with RPMI-1640 medium containing 10% FBS, 100 units/mL penicillin, and 100 μg/mL streptomycin, which were maintained in the incubator with 5% CO_2_ at 37°C. 1 × 10^6^ cells/ml were planted in 6-cm culture plates. Also, the plasmids were transfected into cells using Lipofectamine 2000 (#2173184) (Invitrogen, Carlsbad, CA, United States) according to the manufacturer’s instructions.

### Reagents and Antibodies

The main reagents and antibodies used in this article are as follows: baicalin (>98%) (#B20570) was purchased from Shanghai Yuanye Biological Co., Ltd. Antibodies against AKT (#4691T), p-AKT (#2853T), CDK2 (#2546), CDK4 (#12790), Cyclin E2 (#4132), Bax (#5023T), Bcl-2 (#4223), and α-tubulin (#2125) were purchased from Cell Signaling Technology.

### Cell Viability Assay

The cell viability of H1299 and H1650 with the baicalin treatment was determined using CCK8 (#MA0218) (meilunbio). The 96-well plates were planted were 5 × 10^4^/ml cells per well, which was treated with baicalin (0, 50, and 100 μg/ml) for different times. After treatment, the cell viability was measured at 450 nm wavelength.

### Colony-Formation Assay

A density of 3 × 10^3^/ml cells was planted in 10-cm plates. The cells after being treated with baicalin were then maintained for about 2 weeks until they grew to visible colonies. Then, the colonies were fixed with 4% paraformaldehyde and stained with crystal violet.

### Cell Cycle Analysis by Flow Cytometry

The 6-cm dish was seeded with 5 × 10^5^ cells then given different treatments for 24 h. Then subsequently, the cells were stained using a cell cycle staining kit (#CCS012) purchased from MultiSciences (Lianke) Biotech Co., Ltd., for 30 min in a dark room.

### Apoptosis Assays

The percent of cell apoptosis was assayed using the FITC Annexin V Apoptosis Detection kit according to the instructions (#556547) (BD, United States).

### Western Blotting Analysis

The cells from the control and treatment groups were collected and lysed by RIPA. After being centrifuged at a high speed, the supernatant was collected. The protein concentrations were measured, then the total protein was separated by SDS-PAGE (8–12%) and transferred to a PVDF membrane. The PVDF membrane was blocked, and the primary antibody was incubated. Finally, the secondary antibody was incubated and detected by ECL Prime Western Blotting Reagent (#RPN2232) (Amersham).

### *In vivo* Lung Tumor Mouse Model

Female BALB/c-nu mice were purchased and raised for a week to adapt to the environment, then the subcutaneous tumor model was established by subcutaneous injection of tumor cells with the final concentration at 5 × 10^6^/ml and subcutaneous injection in the underarm. After the tumor was formed, mice were randomly divided into four groups: control group (100 μl, 5% DMSO), baicalin group (100 μl, 20 mg/kg baicalin diluted with 5% DMSO), SC79 group, and SC79 combined use of baicalin group. Mice were treated for 16 days, and then the tumors were collected and fixed. The experiment was approved by the Animal Care and Use Committee of Zhejiang University of Traditional Chinese Medicine (approval ID: 11139).

### Immunohistochemistry

Tumor tissues were fixed with for 24 h with 10% neutral-buffered formalin. Then, tissues were sectioned (4 μm) and sodium citrate buffer was used for the antigen retrieval of sections. Then, the sections were incubated with 10% normal goat serum and primary antibody for 1 h and overnight, respectively. Next, the biotinylated goat anti-rabbit IgG (Vector Laboratories, Burlingame, CA, United States; catalog # BA-1000; dilution 1:200) was used to incubate the sections for 1 h and subsequently incubate with avidin–biotin-horseradish peroxidase (VECTASTAIN^®^ Elite ABC kit; Vector Laboratories; Burlingame, CA, United States; catalog # PK-4010) for 1 h. Finally, color was detected using DAB substrate kit (Vector Laboratories, catalog # SK-4100).

### Statistical Analysis

All tests were performed for three times in all studies unless otherwise stated. All data were expressed as mean ± SD. Student’s *t*-test was employed for the significance calculate between different groups.

## Results

### Baicalin Inhibited the Growth of Lung Cancer Cells

**FIGURE 1:**
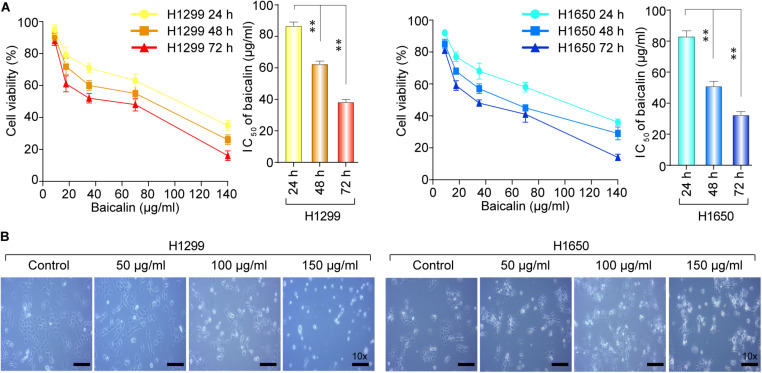
The cell viability of H1299 and H1650 cells was detected after the treatment with baicalin. **(A)** The cell viability was detected with different dose of baicalin treatment for three different time points in H1299 and H1650 lung cancer cells. **(B)** The cell morphological changes were observed by light microscopy with or without treatment of baicalin. The mean ± SD is shown, **P < 0.01.

To test the biological function of baicalin in lung cancer, we first analyzed the cytotoxicity and growth inhibition effects of baicalin on lung cancer cells H1299 and H1650. After the treatment with various concentrations of baicalin at three time points, the results indicated that cell viability of lung cancer cells was inhibited by baicalin and the efficacy was positively correlated with drug dose and treatment time (Figure 1A). Meanwhile, light microscopy observation also showed that baicalin significantly inhibited the viability of these two lung cancer cells compared with their controls (Figure 1B), with more detached and shrunken cells appearing.

### Baicalin Triggered Apoptosis, Inhibited Cell Proliferation, and Blocked Cell Cycle G1/S Transition in Lung Cancer Cells

**FIGURE 2:**
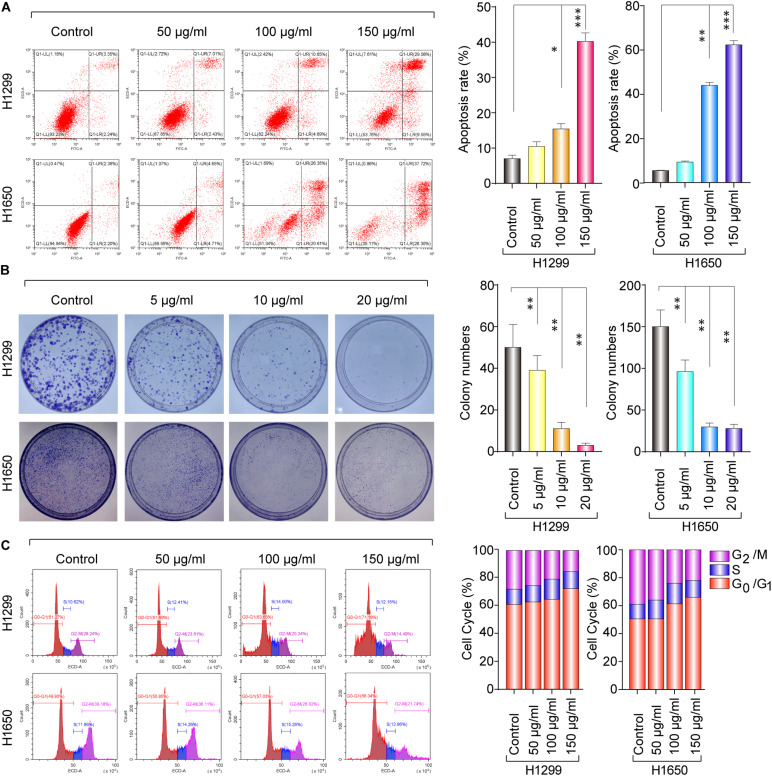
The anticancer effect of baicalin in H1299 and H1650 cells. **(A)** The apoptosis was assayed with or without treatment of baicalin, and representative results were analyzed quantitatively after the treatment. **(B)** The colony-formation assay was performed with or without baicalin treatment, and representative results were analyzed quantitatively. **(C)** The cell cycle was assessed with or without treatment of baicalin by flow cytometry, and representative results were quantitative analyzed. The mean ± SD is shown, *P < 0.05, **P < 0.01, ***P < 0.001.

To obtain the effect of baicalin on apoptosis, the cell viability was detected by flow cytometry. As shown in Figure 2A, the proportion of apoptotic cells significantly increased in the baicalin treatment group, compared with the control. Colony formation assay was used to determine the anti-proliferation effect of baicalin. Moreover, the colony forming ability was remarkably inhibited by baicalin treatment, even with low concentrations such as 5 μg/ml (Figure 2B), indicating that baicalin treatment inhibited cell proliferation of lung cancer. The mechanism in which baicalin inhibited the lung cancer cell proliferation was further investigated via flow cytometry to detect whether baicalin could cause cell cycle arrest. Our data showed that cell cycle was remarkably arrested in the G1/S phase, accompanied with a decreased G2/M phase (Figure 2C). Taken together, we found that the cell-cycle arrest and apoptosis induction might be the major antitumor mechanism of baicalin in lung cancer cells.

**FIGURE 3:**
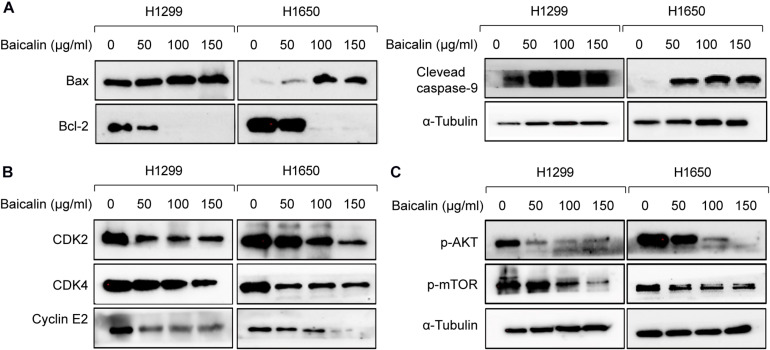
The apoptosis and cell cycle arrest regulatory proteins were analyzed by western blotting. **(A)** The apoptosis-related proteins were analyzed by western blotting in both control and baicalin groups. **(B)** The cell cycle arrest-related proteins were analyzed by western blotting in both control and baicalin groups. **(C)** The protein expressions of Akt and mTOR in lung cancer cells treated with or without baicalin were determined by western blotting.

Furthermore, the results from western blotting in Figure 3A show that the protein expression level of Bax was significantly upregulated in a dose-dependent manner, whereas Bcl-2 expression levels were remarkably decreased. The mitochondrial apoptosis proteins Bax and Bcl-2 can be activated by caspase-9 (Venteo et al., 2010); therefore, caspase-9 expression was also detected by western blotting in both baicalin treatment group and control group. Also, the results showed that activated caspase-9 was remarkably upregulated in a dose-dependent manner after baicalin treatment, when compared with their controls (Figure 3A).

As we know, cell cycle progression is controlled by a cyclin component and several cyclin-dependent kinases (CDKs), including CDK4/6-cyclin D and CDK2-cyclin E (Phan and Croucher, 2020). The above flow cytometric analysis shows that baicalin treatment could induce G1/S phase arrest. Thus, western blotting was performed to detect whether these cell cycle mediators were regulated by baicalin treatment, and we found that the proteins including CDK2, CDK4, and Cyclin E2 were decreased significantly after baicalin treatment (Figure 3B).

### Akt Signal Was Involved in Baicalin-Induced Cell Cycle Arrest and Apoptosis in Lung Cancer Cells

Akt/mTOR is a classical pathway involved in numerous cellular functions, including cellular proliferation, cell cycle progression, and the development of cancer (American Association for Cancer Research, 2017; Janku et al., 2018). In the Akt/mTOR pathway, protein mTOR can be activated by Akt, which can inhibit cellular apoptosis and promote cell proliferation (Zhang et al., 2017).

**FIGURE 4:**
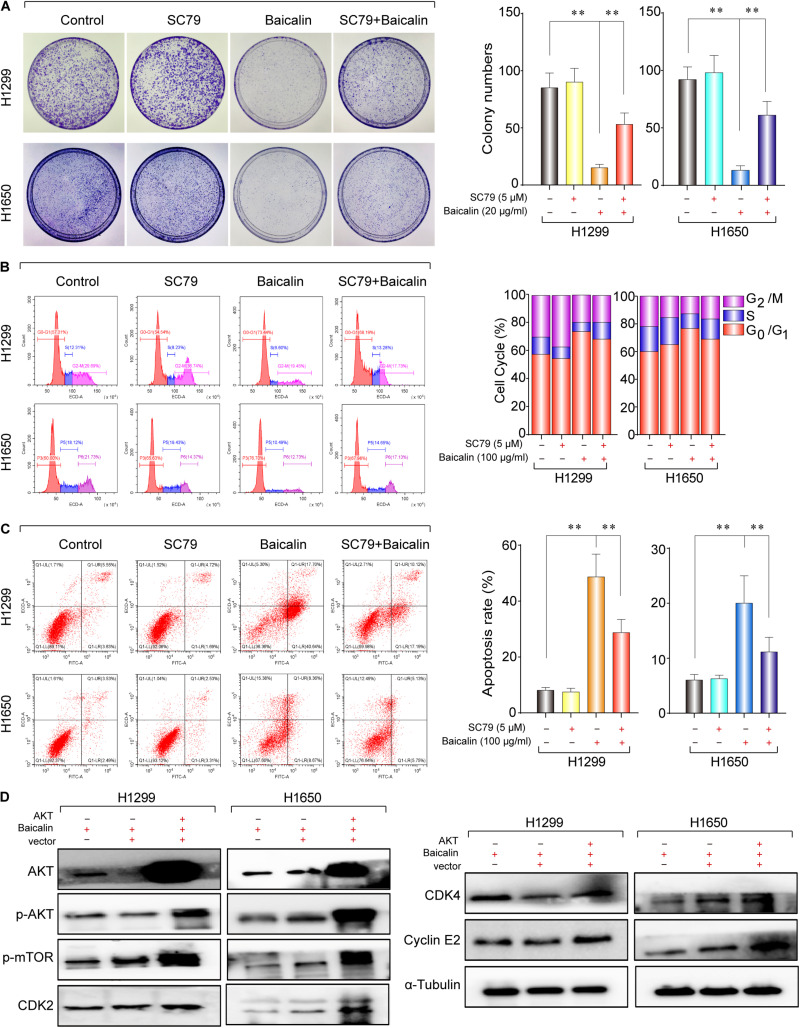
The study of the anticancer mechanism of baicalin in H1299 and H1650 cells. **(A)** The AKT agonist SC79 (5 μM) was used in colony-formation assay to verify the anticancer effect of baicalin, and representative results were analyzed quantitatively after the treatment. **(B)** The AKT agonist SC79 (5 μM) was used to verify the anticancer effect of baicalin by cell cycle assay, and representative results were analyzed quantitatively after the treatment. **(C)** The AKT agonist SC79 (5 μM) was used in apoptosis assay to verify the anticancer effect of baicalin, and representative results were analyzed quantitatively after the treatment. **(D)** The effects of overexpression of AKT on the AKT/mTOR signal pathway and its downstream cell cycle regulatory proteins CDK2, CDK4, and Cyclin E2. The mean ± SD is shown, **P < 0.01.

Hence, the protein expression in the Akt/mTOR pathway was detected by western blotting. As a result, the expression of p-Akt and p-mTOR was remarkably downregulated after baicalin treatment (Figure 3C). To investigate whether the Akt signal was a key determinant for the antitumor activity of baicalin in lung cancer, we specifically enhanced Akt activation using a pharmacological activator SC79 (5 μM) and the Akt overexpression plasmid. As a result, when the Akt activator SC79 was added to baicalin-treated lung cancer cells, the decreased cell proliferation, increased cell cycle arrest, and increased apoptosis were significantly rescued (Figures 4A–C). Next, the Akt overexpression plasmid was used to transfect H1299 and H1650 cells, which was verified by western blotting (Figure 4D). This result showed that the antitumor activity of baicalin was remarkably attenuated by Akt overexpression, followed by upregulated phosphorylation of mTOR and expression of cell cycle-related proteins in the G1/S phase including CDK2, CDK4, and Cyclin E2. In conclusion, our results demonstrate that baicalin has antitumor potential via suppressing the Akt/mTOR pathway *in vitro*.

### Baicalin Exerted Antitumor Efficacy by Suppressing Akt Activity *in vivo*

**FIGURE 5:**
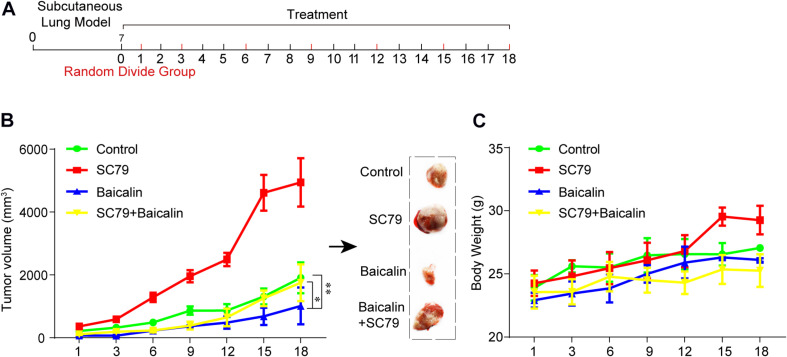
The study of baicalin-suppressed lung cancer growth *in vivo*. **(A)** The scheme of tumor inoculation and systemic injection. **(B)** The tumor volume was monitored in the control group (*n* = 4), SC79 group (*n* = 5), baicalin group (*n* = 5), and SC79 combined use of baicalin group (*n* = 5) to assess the *in vivo* anticancer effect of baicalin. *P < 0.05, **P < 0.01. **(C)** The body weight was monitored in control group, SC79 group, baicalin group, and SC79 combined use of baicalin group to assess the *in vivo* side effect of the baicalin.

The antitumor effect of baicalin was also evaluated *in vivo*. The establishment of the subcutaneous tumor model and drug administration regimen in nude mice is shown in Figure 5A. First, BALB/c nude mice were used to establish the subcutaneous tumor model. When xenografts reach 100 mm^3^, the mice were, respectively, divided into the control group (DMSO), baicalin group (20 mg/kg), SC79 group, and SC79 combined with baicalin group. The data showed that tumor volume was significantly inhibited by baicalin treatment when compared with the control group (Figure 5B). Importantly, there was no significant change in the body weights in four different groups, which indicated that there was no or much lower toxicity when treated with baicalin (Figure 5C).

**FIGURE 6:**
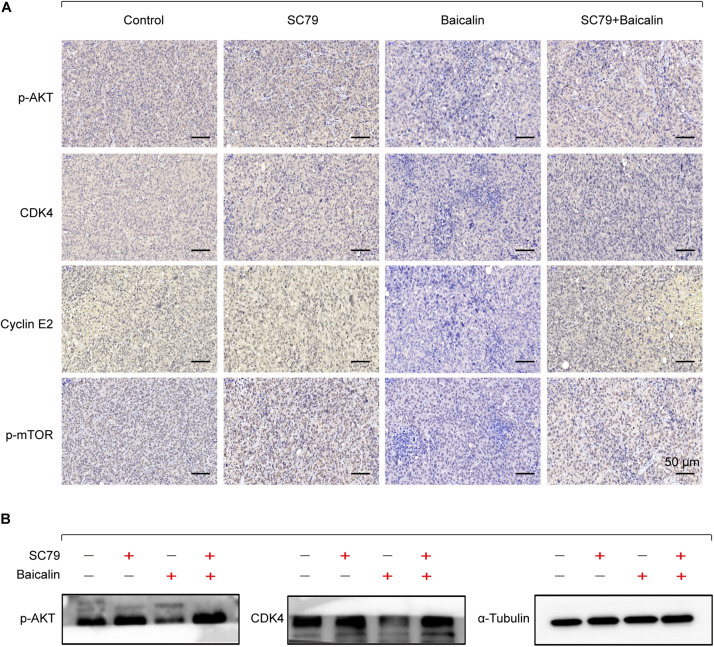
Immunohistochemical staining and western blotting for xenograft tumor sections. **(A)** The immunohistochemistry analysis of the protein expression of p-Akt, p-mTOR, CDK4, and Cyclin E2 in the control group, SC79 group, baicalin group, and SC79 combined use of baicalin group. **(B)** Western blotting was employed to measure the proteins p-Akt and CDK4.

Then, the apoptotic and cell cycle arrest-related proteins from different treatment groups were detected by immunohistochemical staining. As shown in Figure 6A, baicalin treatment caused a significant decrease of p-Akt and p-mTOR as well as proteins CDK4 and Cyclin E2. Importantly, these results were also demonstrated by western blotting (Figure 6B). In the further study, we found that co-treatment with baicalin and Akt activator SC79 significantly attenuated the decreased tumor size and upregulated the expression of p-Akt, p-mTOR, CDK4, and Cyclin E2, when compared with baicalin treatment alone (Figures 5, 6). These results suggest that baicalin could inhibit tumor growth *in vivo* by suppressing cell cycle progression via inhibiting the Akt signal pathway.

## Discussion

Baicalin, as a natural active ingredient derived from the traditional Chinese medicine *Scutellaria baicalensis* Georgi, has great potential to be used for various diseases including cancer. Several studies have reported the role of baicalin in lung cancer. Baicalin might inhibit proliferation of lung cancer cells as a PDZ-binding kinase/T-LAK cell-originated protein kinase (PBK/TOPK) inhibitor both *in vitro* and *in vivo* (Diao et al., 2019). The cell viability, invasion, and metastasis of lung cancer cell A549 and H1299 were detected with the treatment of baicalin by activating the SIRT1/AMPK signaling pathway (You et al., 2018). Baicalin was demonstrated to have cytotoxic and antitumor effects in human chondrosarcoma through inducing apoptotic death and downregulating the phosphoinositide 3-kinase (PI3K)/Akt/mTOR pathway (Zhu et al., 2019). It was also shown that baicalin could suppress cell survival, migration, and invasion of mesothelioma cell lines, while sensitizing the cells to chemotherapeutic agents through inhibiting the PI3K/AKT/mTOR signaling pathway (Xu et al., 2019). These studies indicated that baicalin has great potential to be developed as a new anticancer drug; however, its probable antitumor mechanism in lung cancer has not been elucidated, which has become a great obstacle to its clinical application.

**FIGURE 7:**
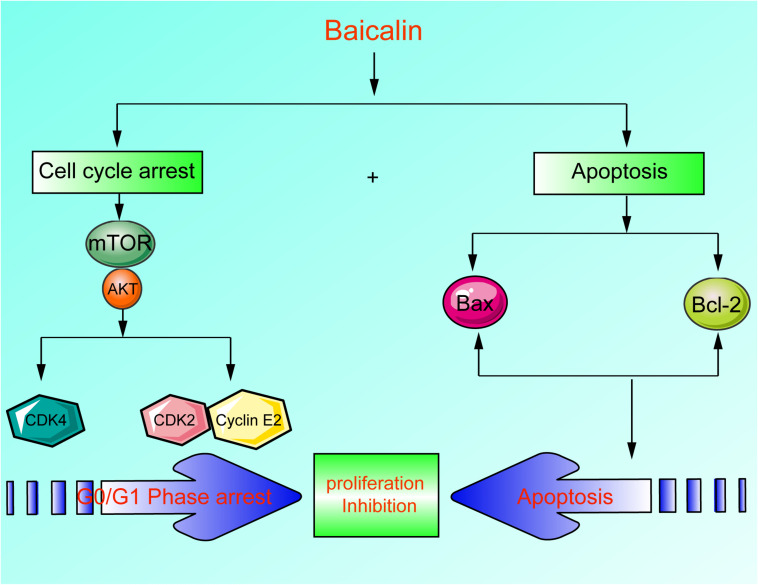
A scheme about the central role of erianin in ferroptosis induction and migration inhibition. Baicalin exerted its anticancer activity through inducing apoptosis, inhibiting cell proliferation, and blocking cell cycle G1-S transition in lung cancer cells in lung cancer cells.

In this study, we reported that the antitumor activity of baicalin was proved through inducing apoptosis, inhibiting cell proliferation, and blocking cell cycle G1/S transition in lung cancer cells (Figure 7). Currently, it has been found that baicalin has multiple mechanisms to induce apoptosis and inhibit proliferation. However, it has not been reported that baicalin can regulate the Akt/mTOR pathway and G1/S cell cycle pathway in lung cancer cells. Therefore, we investigated the relationship between baicalin-induced anti-proliferation effects in lung cancer cells and the Akt/mTOR pathway. As a result, the Akt/mTOR pathway was significantly inhibited after baicalin treatment, accompanied by the attenuation of G1/S phase-regulated proteins including CDK2, CDK4, and Cyclin E2. Next, we showed that Akt activation by using a pharmacological activator SC79 and overexpressed Akt plasmid attenuated baicalin-induced cell cycle arrest and survival inhibition in both *in vitro* and *in vivo* experiments.

## Conclusion

Our data suggest that baicalin exerted its antitumor activity mainly by inducing Akt-dependent cell cycle arrest and promoting apoptosis. This novel information partially explained the anti-proliferation property of baicalin on lung cancer cells and would hopefully provide a new natural compound for lung cancer treatment. However, low bioavailability and poor pharmacokinetics have limited the clinical application of baicalin. Thus, improving the bioavailability and pharmacokinetics will be an important issue in the future.

## Data Availability Statement

The raw data supporting the conclusions of this article will be made available by the authors, without undue reservation.

## Ethics Statement

The animal study was reviewed and approved by 2020128.

## Author Contributions

XS, TX, NK, and XD guided and designed the research. XH and PC performed the cell viability assay, western blotting analysis, and *in vivo* experiment. JF, SL, and TD provided major technical supports. XC, TP, LY, TJ, YX, QG, and CW contributed material information gathering and data analysis. WM, WL, MZ, ZY, RZ, and BC collected and sorted the data. XS and PC wrote the manuscript with contributions from the other authors. All authors contributed to the article and approved the submitted version.

## Conflict of Interest

The authors declare that the research was conducted in the absence of any commercial or financial relationships that could be construed as a potential conflict of interest.
